# Identification of Long Non-Coding RNAs Related to Skeletal Muscle Development in Two Rabbit Breeds with Different Growth Rate

**DOI:** 10.3390/ijms19072046

**Published:** 2018-07-13

**Authors:** Liangde Kuang, Min Lei, Congyan Li, Xiangyu Zhang, Yongjun Ren, Jie Zheng, Zhiqiang Guo, Cuixia Zhang, Chao Yang, Xiuli Mei, Min Fu, Xiaohong Xie

**Affiliations:** 1 Sichuan Animal Sciences Academy, Chengdu 610066, China; happyboy5851258@163.com (L.K.); meets888@163.com (M.L.); licongyan0311@163.com (C.L.); chadance33@163.com (X.Z.); swees2@126.com (Y.R.); laolang1188@163.com (J.Z.); ygzhiq@126.com (Z.G.); dawn713@163.com (C.Z.); yc20040736@163.com (C.Y.); mxl038@126.com (X.M.); safm2017@126.com (M.F.); 2 Animal Breeding and Genetics Key Laboratory of Sichuan Province, Chengdu 610066, China

**Keywords:** rabbit, lncRNA, skeletal muscle development, RNA-Sequencing

## Abstract

Skeletal muscle development plays an important role in muscle quality and yield, which decides the economic value of livestock. Long non-coding RNAs (lncRNAs) have been reported to be associated with skeletal muscle development. However, little is revealed about the function of lncRNAs in rabbits’ muscle development. LncRNAs and mRNAs in two rabbit breeds (ZIKA rabbits (ZKR) and Qixin rabbits (QXR)) with different growth rates at three developmental stages (0 day, 35 days, and 84 days after birth) were researched by transcriptome sequencing. Differentially expressed lncRNAs and mRNAs were identified for two rabbit breeds at the same stages by DESeq package. Co-expression correlation analysis of differentially expressed lncRNAs and mRNAs were performed to construct lncRNA–mRNA pairs. To explore the function of lncRNAs, Gene Ontology (GO) analysis of co-expression mRNAs in lncRNA–mRNA pairs were performed. In three comparisons, there were 128, 109, and 115 differentially expressed lncRNAs, respectively. LncRNAs TCONS_00013557 and XR_518424.2 differentially expressed in the two rabbit breeds might play important roles in skeletal muscle development, for their co-expressed mRNAs were significantly enriched in skeletal muscle development related GO terms. This study provides potentially functional lncRNAs in skeletal muscle development of two rabbit breeds and might be beneficial to the production of rabbits.

## 1. Introduction

The meat of rabbits as a functional food provides dietetic properties and remarkable nutritive value [1,2]. It is becoming more and more popular to people on account of its characteristics of rich protein, low cholesterol, and low fat. Thus, improving the yield and quality of rabbits muscle might be the central task for breeding rabbits.

Most long non-coding RNAs (lncRNAs) generate at certain stages of biological development in a specific manner of cell or tissue. Emerging research showed that lncRNAs participated in the development of skeletal muscle in livestock. For example, Ramayo-Caldas et al. identified 55 differentially expressed lncRNAs between high intramuscular fat tissues and low intramuscular fat tissues in pigs by RNA-Sequencing, suggesting that lncRNAs were related to the fat metabolism of muscle [3]. Billerey et al. found 418 intergenic lncRNAs in all nine muscle samples of Limousin bull calves by RNA-Sequencing and validated 13 intergenic lncRNAs by Real-Time Polymerase Chain Reaction (RT-PCR) [4]. Meanwhile, part intergenic lncRNAs were found located in meat quality traits related loci [4]. Novel lncRNAs identified from chicken skeletal muscle by transcriptome sequencing presented differential expression level in a variety of tissues, and overexpression of lncRNA *Gallus gallus* (gga)-lnc-0181 in skeletal muscle might play a significant role in the muscle development of chicken [5]. Using RNA-Sequencing, several lncRNAs and protein coding genes associated with muscle development were screened in sheep [6]. All researchers above indicated that lncRNAs play important roles in muscle development.

However, there is little research on rabbits’ lncRNAs associated with muscle development. The expression patterns of lncRNAs in the rabbits’ skeletal muscle development remain widely unknown. Thus, we detected the expression patterns of lncRNAs and mRNAs in two rabbit breeds differing in growth rate at three developmental stages (0 day, 35 days, and 84 days after birth). The potential lncRNAs related to muscle development in two different rabbit breeds were predicted according to the function of corresponding co-expressed mRNAs with the lncRNAs. The study will provide potential lncRNAs related to muscle development of rabbits. It will also provide important data for studying the molecular mechanism of different varieties feeding rabbits’ growth difference and promoting the production of the meat rabbits.

## 2. Results

### 2.1. Sample Information

**Figure 1:**
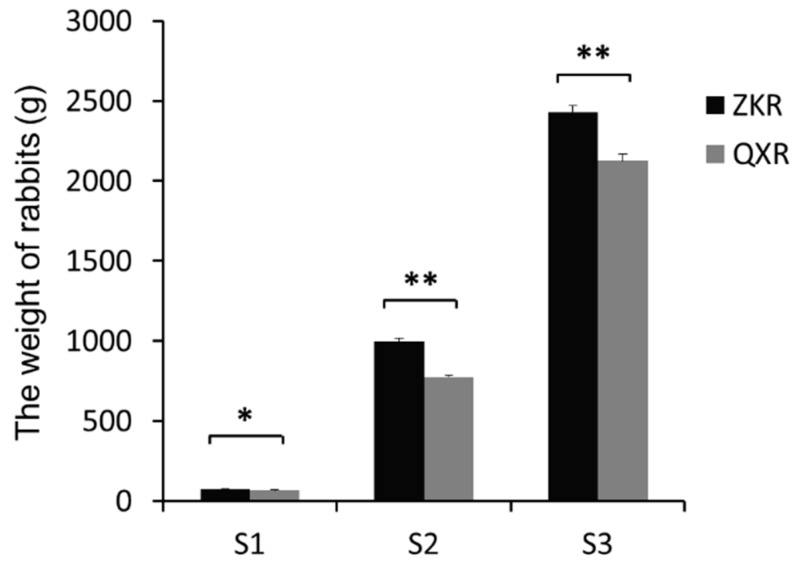
The weight of ZIKA rabbits (ZKR) and Qixin rabbits (QXR) at three development stages. S1, S2, and S3 refer to the age of 0 day, 35 days, and 84 days after birth, respectively. * and ** refer to the statistically significant difference (*p* < 0.05) and extremely significant difference (*p* < 0.001), respectively.

The weight of two rabbit breeds at three developmental stages is shown in Figure 1. The weight of ZIKA rabbits (ZKR) was significantly higher than that of Qixin rabbits (QXR), suggesting that the two kinds of rabbits differed in growth rate and are suited for researching the molecular mechanism of muscle growth and development (all *p* < 0.05).

### 2.2. Reads Filtering and Mapping

The filtering rate of each sample was greater than 90%. The Q30 was not less than 91.4%. After filtering, on average, 95,660,601, 94,297,177, 90,539,959, 97,386,913, 97,460,159, and 91,414,542 clean reads were obtained for three samples each of ZKR_S1, ZKR_S2, ZKR_S3, QXR_S1, QXR_S2, and QXR_S3, respectively, and more than 90% were mapped to the *Oryctolagus cuniculus* reference genome (OryCun2.0) (Table 1). All these results suggested that the data of RNA-Sequencing were quietly credible.

### 2.3. Identification and Characterization of lncRNAs

**Figure 2:**
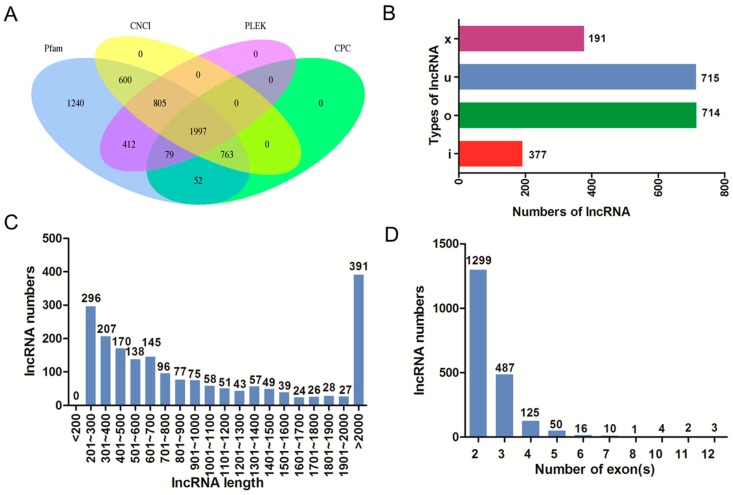
The features of rabbits’ muscle long non-coding RNAs (lncRNAs). (**A**) Venn graph of lncRNA transcripts from coding potential calculator (CPC), coding-non-coding index (CNCI), the protein families database (Pfam), and predictor of long non-coding RNAs and messenger RNAs based on an improved k-mer scheme (PLEK); (**B**) The numbers of four types of lncRNAs including intergenic lncRNA (u), intronic lncRNA (i), anti-sense lncRNA (x), and sense-overlapping lncRNA (o); (**C**) The length distribution of lncRNAs; (**D**) The number of exons per lncRNA.

The intersection of coding potential calculator (CPC), coding-non-coding index (CNCI), the protein families database (Pfam), and predictor of long non-coding RNAs and messenger RNAs based on an improved k-mer scheme (PLEK) results yielded 1997 lncRNA transcripts (Figure 2A). Among these transcripts, there were four types of lncRNAs including intergenic lncRNA (u, 714), intronic lncRNA (i, 191), anti-sense lncRNA (x, 377), and sense-overlapping lncRNA (o, 715) (Figure 2B). LncRNAs with size length >2000 bp accounted for the largest proportion (Figure 2C). Most lncRNAs contained 2 exons (Figure 2D).

### 2.4. Principal Component Analysis (PCA) and Differential Expression Analysis

**Figure 3:**
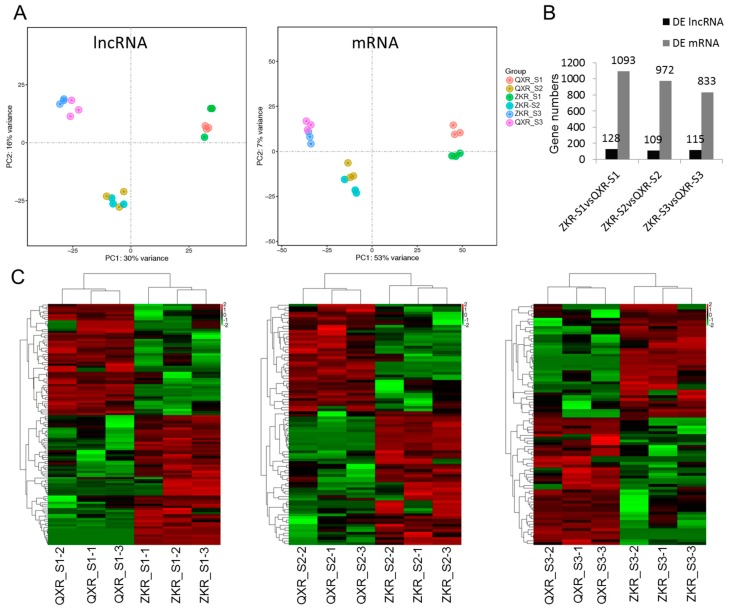
Principal component analysis (**A**), the gene numbers of differentially expressed (DE) lncRNAs and mRNAs (**B**) and heatmaps of differentially expressed lncRNAs (**C**) in three comparisons. ZKR: ZIKA rabbits; QXR: Qixin rabbits; S1, S2, and S3 refer to the age of 0 day, 35 days, and 84 days after birth, respectively.

Both the results of PCA for lncRNAs and mRNAs showed that the samples (ZKR and QXR) with the same stages (S1, S2, and S3) were more similar (Figure 3A). The numbers of differentially expressed lncRNAs and mRNAs between ZKR and QXR at S1, S2, and S3 are shown in Figure 3B. A total of 128, 109, and 115 differentially expressed lncRNAs were identified between ZKR and QXR at S1, S2, and S3, respectively. Heatmaps of differentially expressed lncRNAs in the comparisons of ZKR_S1 vs. QXR_S1, ZKR_S2 vs. QXR_S2, and ZKR_S3 vs. QXR_S3 suggested that the samples of QXR and ZKR with the same stages were distinguished by clustering (Figure 3C). 

### 2.5. lncRNA–mRNA Co-Regulated Pairs

**Figure 4:**
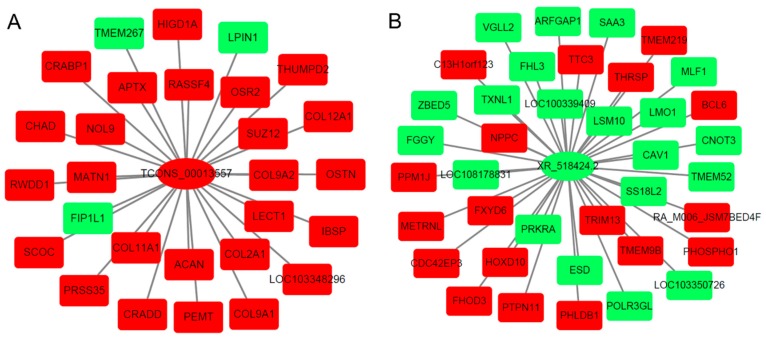
The networks of lncRNA TCONS_00013557 (**A**) and XR_518424.2 (**B**) with the corresponding co-expression mRNAs in each comparison. Green refers to down-regulated gene; red refers to up-regulated gene.

Co-expression correlations of differentially expressed lncRNA and differentially expressed mRNA from each comparison were performed according to the fragments per kilobase per million reads (FPKM). The co-expressed mRNAs in each lncRNA–mRNA co-regulated pair were selected to explore the main functional role of lncRNAs. Figure 4 shows the networks of lncRNAs TCONS_00013557 and XR_518424.2 with the corresponding co-expression mRNAs.

### 2.6. Gene Ontology (GO) Analysis for Co-Expression mRNA of Each lncRNA

**Figure 5:**
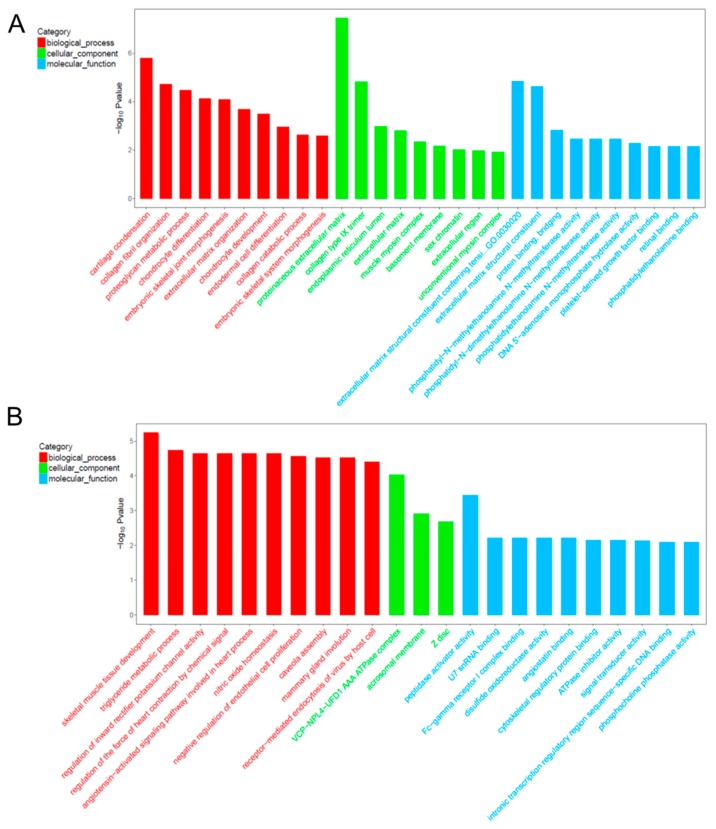
The top Gene Ontology (GO) enrichment analysis of the corresponding co-expression mRNAs of lncRNA TCONS_00013557 (**A**) and XR_518424.2 (**B**).

Based on the GO results, a total of 29 lncRNAs, whose co-expressed mRNAs had the most GO terms and the enriched mRNA ≥5, were selected for three comparisons with the same stages (Table 2). In order to identify muscle development related lncRNAs in rabbits differing in growth rate, lncRNAs whose co-expressed mRNA enriched in the skeletal muscle development related GO terms (embryonic skeletal joint morphogenesis, embryonic skeletal system morphogenesis, and skeletal muscle tissue development) were selected from the 29 lncRNAs. For the comparison of ZKR_S1 vs. QXR_S1, lncRNA TCONS_00013557 was selected because of its co-expressed mRNAs enriching in the GO terms of collagen fibril organization, proteoglycan metabolic process, embryonic skeletal joint morphogenesis, extracellular matrix organization, collagen catabolic process, and embryonic skeletal system morphogenesis on biological process (Figure 5A). As for cellular component, GO terms were dominantly composed of proteinaceous extracellular matrix and extracellular matrix. Within molecular function category, GO terms were significantly composed of extracellular matrix structural constituent conferring tensile strength, extracellular matrix structural constituent, and protein binding. Similarly, lncRNA XR_518424.2 was identified for the comparison of ZKR_S2 vs. QXR_S2 on account of its co-expressed mRNAs mainly enriching in the GO term of skeletal muscle tissue development (Figure 5B). For the comparison of ZKR_S3 vs. QXR_S3, no lncRNAs were selected. The enriched mRNAs of the GO terms are shown in Table 3. Co-expression mRNAs [Odd-skipped related 2 (Osr2), Collagen type II α1 (Col2a1), and Collagen type XI α1 (Col11a1)] of TCONS_00013557 and co-expression mRNAs [Vestigial-like 2 (Vgll2), Caveolin 1 (Cav-1), and Hoxd10] of XR_518424.2 mainly enriched skeletal muscle development related GO terms (Table 3). 

### 2.7. Validation of the Selected lncRNAs and Co-Expression mRNAs

**Figure 6:**
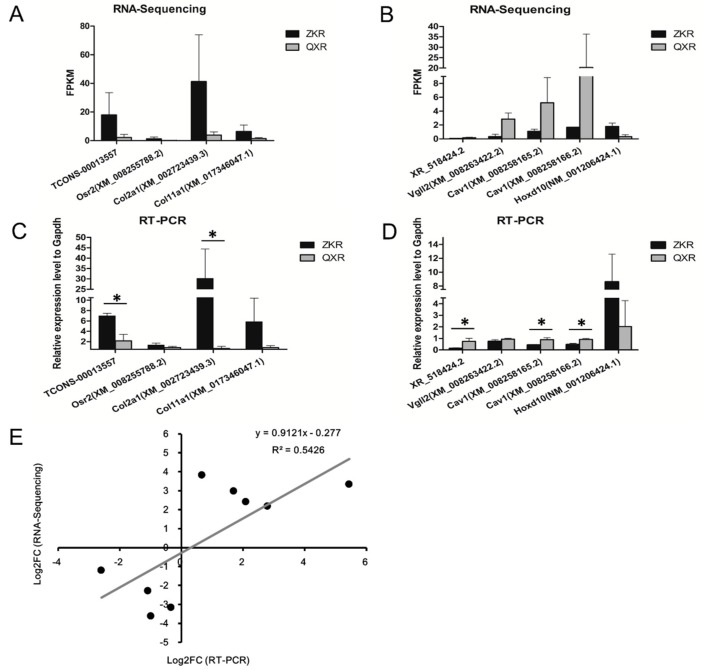
The expression levels of lncRNAs TCONS_00013557 and XR_518424.2 and the corresponding co-expression mRNAs by transcriptome sequencing and RT-PCR. The expression levels of lncRNA TCONS_00013557 and its co-expressed mRNAs (Osr2, Col2a1, and Col11a1) at the stage of S1 by RNA-Sequencing (**A**) and RT-PCR (**C**). The expression levels of XR_518424.2 and its co-expressed mRNAs (Vgll2, Cav1, and Hoxd10) at the stage of S2 by RNA-Sequencing (**B**) and RT-PCR (**D**). (**E**) Linear regression analysis of fold change (FC) data between RT-PCR and RNA-Sequencing. ZKR: ZIKA rabbits; QXR: Qixin rabbits; S1, S2, and S3 refer to the age of 0 day, 35 days, and 84 days after birth, respectively. * refers to the statistically significant difference (*p* < 0.05). Black dots represent log2 transformed FC values of a gene obtained from RT-PCR (*X*-axis) and RNA-Sequencing (*Y*-axis). R: correlation coefficient.

The RNA-Sequencing results of the selected lncRNAs and co-expression mRNAs are shown in Figure 6A,B. The expression levels of the selected genes were validated by RT-PCR. The RT-PCR results confirmed that lncRNA TCONS_00013557, Osr2, Col2a1, and Col11a1 were higher in ZKR than in QXR at S1 (Figure 6C). The lncRNA XR_518424.2 and its co-expressed mRNAs (Vgll2 and Cav1) were all lower in ZKR than in QXR at S2, whereas co-expressed mRNA Hoxd10 was higher expressed in ZKR at S2 by RT-PCR (Figure 6D). All the RT-PCR results were in agreement with the RNA-Sequencing results. Among these genes, TCONS_00013557, Col2a1, XR_518424.2, and Cav1 were significantly differentially expressed between ZKR and QXR. Correlation analysis showed significantly positive correlation in fold change data between RT-PCR and RNA-Sequencing (correlation coefficient *R* = 0.737, *p* < 0.05), confirming our transcriptome sequencing analysis (Figure 6E).

## 3. Discussion

Emerging research suggests that lncRNAs play a significant role in the muscle development of pigs [3], bull calves [4], chickens [5], sheep [6], and humans [7]. Nevertheless, the lncRNAs analysis of rabbits’ muscle has not been studied. In the present work, we identified lncRNAs and mRNAs in muscle tissues of two rabbit breeds by transcriptome sequencing. We explored the rabbits’ muscle lncRNAs from structure and expression level. To reveal the functions, the co-expression correlations of differentially expressed lncRNA and differentially expressed mRNA from each comparison and GO analysis of co-expression mRNAs were performed. 

In this study, a total of 1997 lncRNA transcripts were found. Most lncRNAs were longer than 2000 bp and contained 2 exons. The PCA results of mRNA and lncRNA showed higher similarity in the same development stage between the two rabbit species, implying that the comparison was reasonable. Comparing the QXR to ZKR, 128, 109, and 115 differentially expressed lncRNAs were identified between ZKR and QXR at S1, S2, and S3, respectively. 

There is a vital interplay between muscle cells and the extracellular matrix in skeletal muscle development [8]. The extracellular matrix, which was mainly composed of collagens, proteoglycans, and glycoproteins, maintained the integrity of skeletal muscle [9]. While inhibiting collagen synthesis, myoblasts differentiation in vitro was blocked [10,11]. Proteoglycans can regulate collagen fibrillogenesis and suppress cell growth [12]. Melo et al. revealed that proteoglycans were essential for skeletal muscle differentiation [13]. Interestingly, co-expression mRNAs of TCONS_00013557 significantly enriched in the GO terms of collagen and proteoglycans related GO terms, indicating that co-expression mRNAs and the corresponding lncRNA TCONS_00013557 affect skeletal muscle development via altering the formation of collagens and proteoglycans in extracellular matrix in development of stage 1.

Co-expression correlation analysis combining GO analysis showed that co-expression mRNAs (Osr2, Col2a1, and Col11a1) of TCONS_00013557, which was differentially expressed between QXR_S1 and ZKQ_S1, significantly enriched in the GO terms such as collagen fibril organization, proteoglycan metabolic process, embryonic skeletal joint morphogenesis, and embryonic skeletal system morphogenesis. Osr2, a zinc-finger transcription factor, was expressed in numerous murine tissues including skeletal muscle tissues and had transcriptional activity involving in postnatal development [14,15]. Col2a1 was expressed in human rotator cuff-derived mesenchymal stem cells, which might be a cell source for muscle repair [16]. Its mRNA level was increased throughout the process of chondrogenic differentiation [17]. Col11a1 was regulated by a transcription activator FP9C, which was associated with cell differentiation in myoblasts and osteoblasts [18]. Consistent with the reports above, these co-expressed mRNAs with lncRNA TCONS_00013557 were all expressed higher in ZKR than in QXR at S1, suggesting that differentially expressed lncRNA TCONS_00013557 was likely involved in the skeletal muscle development of rabbits with different growth rates.

Co-expression mRNAs (Vgll2, Cav-1, and Hoxd10) of XR_518424.2 differentially expressed between QXR_S2 and ZKQ_S2 mainly enriched in skeletal muscle tissue development, in which Vgll2, Cav-1, and Hoxd10 were significantly enriched. Vgll2 played an important role in skeletal muscle differentiation and development myotubes. It was a cofactor of transcription enhancer factor 1 and myocyte enhancer factor 2 [19,20]. Vgll2 expression was skeletal muscle-specific in mammalian adult tissues and increased in differential myotubes [19]. Similarly, Vgll-2 was expressed in different sites of chick skeletal myogenesis and related to skeletal muscle differentiation as downstream gene of myogenic factor [21]. Vgll2 defecting resulted in an increase in fast-twitch fibers’ numbers and Myh7 down-regulated in mice, suggesting that Vgll2 might be related to slow muscle fibers’ programing [22]. Cav-1 was detected in various adult monkey tissues, including skeletal muscle, and was co-located with dystrophin on sarcolemma by immunohistochemistry [23]. Cav-1 was highly expressed in masticatory muscles of murine X-linked muscular dystrophy, which was with muscle injury and progressive muscle weakness caused by lack of dystrophin [24]. Hoxd10 was found differentially expressed in the back muscle of a mouse, absented Myf5, and a regulator for motor neuron development [25]. For all above, low expression of Vgll2 and Cav-1 might promote muscle development; differentially expressed Hoxd10 was related to muscle development. In this work, we found that the expression of Vgll2 and Cav-1 were lower in ZKR than in QXR at S2, whereas higher expression of Hoxd10 was found in ZKR than in QXR at S2. These mRNAs related to skeletal muscle development were co-expressed with the lncRNA XR_518424.2. Thus, XR_518424.2 probably participates in skeletal muscle development of rabbits with different growth rates.

In conclusion, we identified several lncRNAs and co-expressed genes related to skeletal muscle development in two rabbit breeds differing in growth rates. The co-expressed genes were mainly enriched in skeletal muscle development related GO terms. The lncRNAs (TCONS_00013557 and XR_518424.2) and co-expressed genes (Col2a1 and Cav-1) were validated to differentially expressed genes significantly by RT-PCR, confirming the important role of themselves and corresponding lncRNAs. This work provides candidate lncRNAs that may be used to explore the function of lncRNAs in the muscle development of rabbits. Further studies should be performed to validate the function and analyze the mechanism in detail.

## 4. Materials and Methods

### 4.1. Sample Collection

The meat rabbits of used in the experiments—German ZIKA rabbits (ZKR) and Sichuan native Qixin Rabbits (QXR)—were obtained from the rabbit farms of Sichuan Animal Sciences Academy in Chengdu, Sichuan, China. All rabbits used (all were male and belonged to the same family in each breed) were raised under the condition with the same diet and environmental temperature and given free access to water and food. The weight of each rabbit was recorded before longissimus muscle tissues were collected. The longissimus muscle tissues were collected from the ZKR and QXR at the age of 0 day (S1), 35 days (S2), and 84 days (S3) after birth (*n* = 3 for each stage and for each breed), respectively, and saved in liquid nitrogen immediately. The experiment was conducted according to the National Institutes of Health (NIH) Guidelines and National Research Council’s publication “Guide for Care and Use of Laboratory Animals”. The experiment was approved by the Animal Care and Use Committee of the Sichuan Animal Sciences Academy. The identification number was not required since the commercial animal sampling was approved. The application form for welfare and ethical review in animal experimentation was approved by the Sichuan Animal Sciences Academy (the approval date: 22 March 2017).

### 4.2. RNA Isolation, Library Construction, and Sequencing

Total RNA of the longissimus muscle tissues were extracted with Trizol regent (Invitrogen, Carlsbad, CA, USA) and quality qualified RNA were treated with TruSeq Stranded Total RNA with Ribo-Zero Gold kit (Illumina, San Diego, CA, USA) to eliminate the ribosomal RNA. Strand-specific RNA-seq (ssRNA-seq) libraries were prepared according the manufacturer’s instructions using the Illumina Standard RNA sample library preparation kit (Illumina, San Diego, CA, USA). After quantification using the Agilent 2100 bioanalyzer (Agilent, Santa Clara, CA, USA), the strand-specific libraries were sequenced on an Illumina HiSeq X ten instrument that generated paired-end reads of 150 nucleotides. Library construction and Illumina sequencing were performed by OE Biotech CO., LTD (Shanghai, China). The raw data have been deposited in the Sequence Read Archive database at the NCBI under the accession number SRP150254.

### 4.3. Raw Reads Preprocessing

Quality control of the raw reads was completed with Trimmomatic (version 0.36, available online: http://www.mybiosoftware.com/trimmomatic-0-30-flexible-read-trimming-tool-illumina-ngs-data.html) [26] software by the following steps: (1) removing adaptor sequence; (2) removing low quality reads; and (3) eliminating the reads smaller than 50 bases after removing part sequence of reads containing base N (unsure of the base information). Then, the original amount of sequencing, effective quantity of sequencing, Q30 and Guanine and Cytosine content were counted and used to evaluate the quality comprehensively. The qualified reads were mapped to the *Oryctolagus cuniculus* reference genome (OryCun2.0, available online: https://www.ncbi.nlm.nih.gov/genome/?term=OryCun2.0) from NCBI by hisat2 (version 2.1.0, available online: http://www.ccb.jhu.edu/software/hisat).) [27]; the download link of *Oryctolagus cuniculus* reference genome is available online: ftp://ftp.ncbi.nlm.nih.gov/genomes/all/GCF/000/003/625/GCF_000003625.3_OryCun2.0/GCF_000003625.3_OryCun2.0_genomic.gff.gz. 

### 4.4. Prediction of lncRNA and mRNA

We reconstructed the transcription of each sample based on probability model with StringTie (version 1.3.3b.Linux_x86_64, available online: http://ccb.jhu.edu/software/stringtie) software [28] and merged them with Cuffcompare software (version v2.2.1, available online: http://cole-trapnell-lab.github.io/cufflinks). To identify credible lncRNA candidates, we compared the merged transcripts to reference transcripts using Cuffcompare software and reserved four kinds of transcripts called “x” (exonic overlap with reference on the opposite strand), “u” (unknown, intergenic transcript), “o” (generic exonic overlap with a reference transcript), and “i” (a transfrag falling entirely within a reference intron). Then the reserved transcripts were filtered at following criteria: (1) selecting the transcripts with length >200 bp and exon numbers ≥2; (2) predicting the protein-coding-ability with the software of coding potential calculator (CPC, version 0.9-r2, available online: http://CPC.cbi.pku.edu.cn) [29], coding-non-coding index (CNCI, version 1.0, available online: https://github.com/www-bioinfo-org/CNCI) [30], the protein families database (Pfam, version 30.0, available online: http://pfam.xfam.org/) [31], and predictor of lncRNAs and messenger RNAs based on an improved k-mer scheme (PLEK, version 1.2, available online: https://sourceforge.net/projects/plek/files/) [32], respectively, and eliminating the transcripts with protein-coding-ability for each software; the intersection of CPC, CNCI, Pfam, and PLEK results were selected. The expression of samples were calculated with algorithm of FPKM [33]. The expression abundance of transcripts was counted by the method of sequence similarity comparison with software of Bowtie 2 (version 2.2.9, available online: https://sourceforge.net/projects/bowtie-bio/files/bowtie2/2.2.9/) [34] and eXpress (version 1.5.1) [35].

### 4.5. PCA and Differential Expression Analysis of lncRNAs and mRNAs

PCA was employed to explore the correlation among samples according to the expression level of lncRNAs and mRNAs, respectively. The differentially expressed lncRNA or mRNA for three comparisons (ZKR_S1 vs. QXR_S1, ZKR_S2 vs. QXR_S2, and ZKR_S3 vs. QXR_S3) were performed with the DESeq package (version 1.18.0, available online: http://www.bioconductor.org/packages/release/bioc/html/DESeq.html), respectively. To control the false discovery rate, the *p*-value was adjusted by Benjamini and Hochberg’s approach. The lncRNAs or mRNAs with the adjusted *p*-value < 0.05 and |log2(fold change)| > 1 were considered as differentially expressed genes.

### 4.6. Co-Expression Correlations of Differentially Expressed lncRNA and mRNA

To explore the functional role of lncRNA, the co-expression correlations of differentially expressed lncRNA and differentially expressed mRNA from each comparison were performed according to the FPKMs. Then the lncRNA–mRNA co-regulated pairs (Pearson’s correlation coefficient >0.8 and *p*-value < 0.05) were screened for Gene Ontology (GO) analysis.

### 4.7. GO Enrichment Analysis

To explore the main functional role of lncRNAs in the muscle development of rabbits, the mRNAs in all lncRNA–mRNA co-regulated pairs were annotated by GO for differentially expressed lncRNAs. The GO terms with *p*-value < 0.05 were considered as significantly enriched. The top 10 lncRNAs whose co-expressed mRNAs had the most GO terms and the enriched mRNA ≥ 5 were screened. The GO enrichment graphs were drawn for the co-expressed mRNA of the selected lncRNAs in these selected lncRNAs.

### 4.8. RT-PCR

One microgram RNA was transcribed to cDNA. RT-PCR was determined using SYBR-Green PCR master mix kit (Applied Biosystems, Inc., Foster City, CA, USA) and performed on an ABI QuantStudio™ 6 Flex System (Applied Biosystems, Inc., Foster City, CA, USA) with the amplification conditions: one cycle of 95 °C for 10 min, followed by 45 cycles of 95 °C for 15 s, 60 °C for 60 s, and 95 °C for 15 s. The primers for amplication of genes and the internal control Gapdh are shown in Table 4. Three independent experiments were employed to detect the relative expression level. The relative expression level was calculated as below: relative quantification = 2^−ΔΔ*C*t^.

### 4.9. Statistical Analysis

The statistical significance was analyzed by the software of SPSS 21.0 (IBM Corp., Armonk, NY, USA). The experiment data was provided as mean value ± standard deviation. Difference between the groups was analyzed with one-way analysis of variance. *p* < 0.05 and *p* < 0.001 refer to the statistically significant difference (*) and extremely significant difference (**), respectively. The Pearson correlation analysis was performed to evaluate the fold change data between RT-PCR and RNA-Sequencing.

## Figures and Tables

**Table 1:** The results of RNA-Sequencing and clean reads mapping to the reference genome for each group.

Sample	ZKR ^a^_S1 ^b^	ZKR_S2 ^b^	ZKR_S3 ^b^	QXR ^c^_S1	QXR_S2	QXR_S3
Raw reads	100,481,781	99,812,614	99,445,825	100,002,793	99,887,525	99,071,615
Clean reads	95,660,601	94,297,177	90,539,959	97,386,913	97,460,159	91,414,542
Filtering rate	93.26%	92.81%	90.31%	95.51%	95.85%	91.25%
Q30	92.25%	91.40%	95.96%	94.52%	95.74%	96.03%
Total mapped reads	87,463,758 (91.41%)	87,557,832 (92.85%)	83,122,460 (91.81%)	89,812,801 (92.22%)	90,465,752 (92.82%)	83,950,965 (91.84%)
Multiple mapped	10,182,952 (10.65%)	11,506,609 (12.20%)	11,988,424 (13.24%)	10,370,562 (10.65%)	11,019,503 (11.30%)	13,298,066 (14.53%)
Uniquely mapped	77,280,806 (80.77%)	76,051,223 (80.65%)	71,134,037 (78.56%)	79,442,240 (81.58%)	79,446,249 (81.52%)	70,652,899 (77.31%)
Reads map to ‘+’	38,609,153 (40.35%)	37,967,782 (40.26%)	35,526,529 (39.24%)	39,685,378 (40.75%)	39,665,757 (40.70%)	35,482,024 (38.82%)
Reads map to ‘−’	38,671,654 (40.42%)	38,083,441 (40.39%)	35,607,508 (39.33%)	39,756,861 (40.83%)	39,780,492 (40.82%)	35,170,875 (38.49%)

^a^ ZKR: ZIKA rabbits; ^b^ S1, S2, and S3 refer to the age of 0 day, 35 days, and 84 days after birth, respectively; ^c^ QXR: Qixin rabbits.

**Table 2:** The selected lncRNAs with the most Gene Ontology (GO) terms and the enriched mRNA ≥ 5 in each comparison.

Comparison	LncRNA Name
ZKR ^a^_S1 ^b^ vs. QXR ^c^_S1	TCONS_00013557, TCONS_00014076, TCONS_00018134, XR_515577.1, XR_519108.2, XR_519249.1, XR_519800.2, XR_001792901.1, XR_001795022.1
ZKR_S2 ^b^ vs. QXR_S2	TCONS_00013141, TCONS_00018134, TCONS_00031283, TCONS_00034998, TCONS_00036781, XR_518424.2, XR_518559.2, XR_519023.2, XR_001792558.1, XR_001795599.1
QXR_S3 ^b^ vs. ZKR_S3	TCONS_00008020, TCONS_00015535, TCONS_00035456, XR_515521.2, XR_517087.2, XR_519431.2, XR_001792689.1, XR_001792882.1, XR_001794410.1, XR_001795042.1

^a^ ZKR: ZIKA rabbits; ^b^ S1, S2, and S3 refer to the age of 0 day, 35 days, and 84 days after birth, respectively; ^c^ QXR: Qixin rabbits.

**Table 3:** GO terms for co-expressed mRNAs of lncRNAs TCONS_00013557 and XR_518424.2.

Term ^a^ ID	Term Description	Gene Symbols	*p*-Value	FDR ^b^
GO terms for co-expressed mRNAs of TCONS_00013557				
GO:0001502	cartilage condensation	ACAN; COL11A1; COL2A1	1.62 × 10^−6^	0.000197
GO:0030199	collagen fibril organization	ACAN; COL11A1; COL2A1	1.90 × 10^−5^	0.001158
GO:0006029	proteoglycan metabolic process	COL11A1; COL2A1	3.35 × 10^−5^	0.001362
GO:0002062	chondrocyte differentiation	MATN1; COL2A1; OSR2	7.32 × 10^−5^	0.002015
GO:0060272	embryonic skeletal joint morphogenesis	OSR2; COL2A1	8.26 × 10^−5^	0.002015
GO:0030198	extracellular matrix organization	COL9A1; COL11A1; IBSP; COL2A1	0.0002075	0.004219
GO:0002063	chondrocyte development	ACAN; COL11A1	0.0003181	0.005543
GO:0035987	endodermal cell differentiation	COL11A1; COL12A1	0.0011231	0.017127
GO:0030574	collagen catabolic process	COL11A1; COL2A1	0.0023463	0.031805
GO:0048704	embryonic skeletal system morphogenesis	COL11A1; OSR2	0.0026094	0.031835
GO:0005578	proteinaceous extracellular matrix	MATN1; COL12A1; COL9A2; LECT1; ACAN; COL9A1; CHAD	3.53 × 10^−8^	1.06 × 10^−6^
GO:0005594	collagen type IX trimer	COL9A1; COL9A2	1.49 × 10^−5^	0.000224
GO:0005788	endoplasmic reticulum lumen	COL9A1; COL11A1; COL2A1	0.0010591	0.010591
GO:0031012	extracellular matrix	COL12A1; IBSP; COL2A1	0.0015573	0.01168
GO:0005859	muscle myosin complex	LOC103348296	0.0044683	0.02681
GO:0005604	basement membrane	ACAN; COL2A1	0.0068787	0.034393
GO:0001739	sex chromatin	SUZ12	0.0096571	0.039576
GO:0005576	extracellular region	PRSS35; COL11A1; COL9A1; IBSP; COL2A1	0.0106248	0.039576
GO:0016461	unconventional myosin complex	LOC103348296	0.0118729	0.039576
GO:0030020	extracellular matrix structural constituent conferring tensile strength	COL9A1; COL2A1	1.45 × 10^−5^	0.000439
GO:0005201	extracellular matrix structural constituent	MATN1; COL11A1; ACAN	2.37 × 10^−5^	0.000439
GO:0030674	protein binding, bridging	CRADD; COL11A1	0.0015134	0.018665
GO:0000773	phosphatidyl-*N*-methylethanolamine *N*-methyltransferase activity	PEMT	0.0035077	0.021631
GO:0080101	phosphatidyl-*N*-dimethylethanolamine *N*-methyltransferase activity	PEMT	0.0035077	0.021631
GO:0004608	phosphatidylethanolamine *N*-methyltransferase activity	PEMT	0.0035077	0.021631
GO:0033699	DNA 5′-adenosine monophosphate hydrolase activity	APTX	0.0052572	0.023118
GO:0048407	platelet-derived growth factor binding	COL2A1	0.0070038	0.023118
GO:0016918	retinal binding	CRABP1	0.0070038	0.023118
GO:0008429	phosphatidylethanolamine binding	PEMT	0.0070038	0.023118
GO terms for co-expressed mRNAs of XR_518424.2				
GO:0007519	skeletal muscle tissue development	VGLL2; CAV1; HOXD10; CAV1	5.64 × 10^−6^	0.000954
GO:0006641	triglyceride metabolic process	CAV1; PTPN11; CAV1	1.78 × 10^−5^	0.000954
GO:1901979	regulation of inward rectifier potassium channel activity	CAV1; CAV1	2.25 × 10^−5^	0.000954
GO:0003057	regulation of the force of heart contraction by chemical signal	CAV1; CAV1	2.25 × 10^−5^	0.000954
GO:0086098	angiotensin-activated signaling pathway involved in heart process	CAV1; CAV1	2.25 × 10^−5^	0.000954
GO:0033484	nitric oxide homeostasis	CAV1; CAV1	2.25 × 10^−5^	0.000954
GO:0001937	negative regulation of endothelial cell proliferation	CAV1; LOC100339409; CAV1	2.69 × 10^−5^	0.000954
GO:0070836	caveola assembly	CAV1; CAV1	3.00 × 10^−5^	0.000954
GO:0060056	mammary gland involution	CAV1; CAV1	3.00 × 10^−5^	0.000954
GO:0019065	receptor-mediated endocytosis of virus by host cell	CAV1; CAV1	3.86 × 10^−5^	0.000954
GO:0034098	VCP-NPL4-UFD1 AAA ATPase complex	CAV1; CAV1	9.36 × 10^−5^	0.004681
GO:0002080	acrosomal membrane	CAV1; CAV1	0.001231186	0.03078
GO:0030018	Z disc	FHL3; FHOD3; RA_M006_JSM7BED4F	0.002062968	0.034383
GO:0016504	peptidase activator activity	CAV1; CAV1	0.000356053	0.023856
GO:0071209	U7 snRNA binding	LSM10	0.006180344	0.045963
GO:0034988	Fc-gamma receptor I complex binding	RA_M006_JSM7BED4F	0.006180344	0.045963
GO:0015036	disulfide oxidoreductase activity	TXNL1	0.006180344	0.045963
GO:0043532	angiostatin binding	LOC100339409	0.006180344	0.045963
GO:0005519	cytoskeletal regulatory protein binding	CDC42EP3	0.007206806	0.045963
GO:0042030	ATPase inhibitor activity	LOC100339409	0.007206806	0.045963
GO:0004871	signal transducer activity	TRIM13; RA_M006_JSM7BED4F; TMEM9B	0.007345729	0.045963
GO:0001161	intronic transcription regulatory region sequence-specific DNA binding	BCL6	0.008232242	0.045963
GO:0052731	phosphocholine phosphatase activity	PHOSPHO1	0.008232242	0.045963

^a^ Term: GO term or pathway term; ^b^ FDR: false discovery rate.

**Table 4:** Real-time PCR primers sequence.

Gene	Sequence	Annealing Temperature (°C)	Aim Band Length (bp)
TCONS_00013557	F 5′ GCTGCTGCCCTTGGACCTT 3′	60	58
TCONS_00013557	R 5′ CGTCACCCACAAACAGAGCA 3′		
Osr2 (XM_008255788.2)	F 5′ GCACACCCAGACCTCGCCG 3′	60	101
Osr2 (XM_008255788.2)	R 5′ AACAACACGTAGAAAATAGCCCG 3′		
Col2a1 (XM_002723439.3)	F 5′ CATGAGGGCGCGGTAGAGA 3′	60	193
Col2a1 (XM_002723439.3)	R 5′ CTTTGGTCCTGGTTTCCGG 3′		
Col11a1 (XM_017346047.1)	F 5′ CTGGATCCAATGAGATAAATGGC 3′	60	104
Col11a1 (XM_017346047.1)	R 5′ CCCTGGTGGTCCTTCAACAA 3′		
XR_518424.2	F 5′ ACCCTAGTAATTCAGCCTGCTCT 3′	60	140
XR_518424.2	R 5′ TGAGTGGTGAGGGAATGGAATA 3′		
Vgll2 (XM_008263422.2)	F 5′ TCAGCGTGGACTCAGCTCGT 3′	60	135
Vgll2 (XM_008263422.2)	R 5′ CACGAAGTGAGAGGCACAGATG 3′		
Cav1 (XM_008258165.2)	F 5′ TGGGAACGACCTGAGGGTG 3′	60	56
Cav1 (XM_008258165.2)	R 5′ AGTGTAGAGATGTCCCTGCACCA		
Cav1 (XM_008258166.2)	F 5′ TGAGCGGCCGCTGTCGA 3′	60	113
Cav1 (XM_008258166.2)	R 5′ ACTTGCTTCTCGTTCACCTCG 3′		
Hoxd10 (NM_001206424.1)	F 5′ AAGGAAAGCAAAGAGGAAATCAAG 3′	60	106
Hoxd10 (NM_001206424.1)	R 5′ CCAGCGTTTGGTGCTTAGTGT 3′		
Gapdh	F 5′ AGGTCGGAGTGAACGGATTTG 3′	60	60
Gapdh	R 5′ AGTTAAAAGCAGCCCTGGTGAC 3′		

## References

[1] Dalle Zotte A., Szendro Z. (2011). The role of rabbit meat as functional food. Meat Sci..

[2] Hermida M., Gonzalez M., Miranda M., Rodriguez-Otero J.L. (2006). Mineral analysis in rabbit meat from galicia (NW Spain). Meat Sci..

[3] Ramayo-Caldas Y., Mach N., Esteve-Codina A., Corominas J., Castello A., Ballester M., Estelle J., Ibanez-Escriche N., Fernandez A.I., Perez-Enciso M. (2012). Liver transcriptome profile in pigs with extreme phenotypes of intramuscular fatty acid composition. BMC Genom..

[4] Billerey C., Boussaha M., Esquerre D., Rebours E., Djari A., Meersseman C., Klopp C., Gautheret D., Rocha D. (2014). Identification of large intergenic non-coding RNAs in bovine muscle using next-generation transcriptomic sequencing. BMC Genom..

[5] Li T., Wang S., Wu R., Zhou X., Zhu D., Zhang Y. (2012). Identification of long non-protein coding RNAs in chicken skeletal muscle using next generation sequencing. Genomics.

[6] Ren C., Deng M., Fan Y., Yang H., Zhang G., Feng X., Li F., Wang D., Wang F., Zhang Y. (2017). Genome-wide analysis reveals extensive changes in lncRNAs during skeletal muscle development in Hu sheep. Genes.

[7] Matsumoto A., Pasut A., Matsumoto M., Yamashita R., Fung J., Monteleone E., Saghatelian A., Nakayama K.I., Clohessy J.G., Pandolfi P.P. (2017). mTORC1 and muscle regeneration are regulated by the LINC00961-encoded SPAR polypeptide. Nature.

[8] Kjaer M. (2004). Role of extracellular matrix in adaptation of tendon and skeletal muscle to mechanical loading. Physiol. Rev..

[9] Nishimura T. (2015). Role of extracellular matrix in development of skeletal muscle and postmortem aging of meat. Meat Sci..

[10] Nandan D., Clarke E.P., Ball E.H., Sanwal B.D. (1990). Ethyl-3,4-dihydroxybenzoate inhibits myoblast differentiation: Evidence for an essential role of collagen. J. Cell Biol..

[11] Saitoh O., Periasamy M., Kan M., Matsuda R. (1992). Cis-4-hydroxy-l-proline and ethyl-3,4-dihydroxybenzoate prevent myogenesis of C2C12 muscle cells and block myod1 and myogenin expression. Exp. Cell Res..

[12] Velleman S.G. (1999). The role of the extracellular matrix in skeletal muscle development. Poult. Sci..

[13] Melo F., Carey D.J., Brandan E. (1996). Extracellular matrix is required for skeletal muscle differentiation but not myogenin expression. J. Cell. Biochem..

[14] Kawai S., Michikami I., Kitagaki J., Hashino E., Amano A. (2013). Expression pattern of zinc-finger transcription factor odd-skipped related 2 in murine development and neonatal stage. Gene Expr. Patterns.

[15] Kawai S., Kato T., Inaba H., Okahashi N., Amano A. (2005). Odd-skipped related 2 splicing variants show opposite transcriptional activity. Biochem. Biophys. Res. Commun..

[16] Tsai C.C., Huang T.F., Ma H.L., Chiang E.R., Hung S.C. (2013). Isolation of mesenchymal stem cells from shoulder rotator cuff: A potential source for muscle and tendon repair. Cell Transplant..

[17] Okada K., Fukai A., Mori D., Hosaka Y., Yano F., Chung U.I., Kawaguchi H., Tanaka S., Ikeda T., Saito T. (2014). Identification of SCAN domain zinc-finger gene ZNF449 as a novel factor of chondrogenesis. PLoS ONE.

[18] Kinoshita A., Greenwel P., Tanaka S., Di Liberto M., Yoshioka H., Ramirez F. (1997). A transcription activator with restricted tissue distribution regulates cell-specific expression of α1(XI) collagen. J. Biol. Chem..

[19] Maeda T., Chapman D.L., Stewart A.F. (2002). Mammalian vestigial-like 2, a cofactor of TEF-1 and MEF2 transcription factors that promotes skeletal muscle differentiation. J. Biol. Chem..

[20] Chen H.H., Maeda T., Mullett S.J., Stewart A.F. (2004). Transcription cofactor VGL-2 is required for skeletal muscle differentiation. Genesis.

[21] Bonnet A., Dai F., Brand-Saberi B., Duprez D. (2010). Vestigial-like 2 acts downstream of myod activation and is associated with skeletal muscle differentiation in chick myogenesis. Mech. Dev..

[22] Honda M., Hidaka K., Fukada S.I., Sugawa R., Shirai M., Ikawa M., Morisaki T. (2017). Vestigial-like 2 contributes to normal muscle fiber type distribution in mice. Sci. Rep..

[23] Hagiwara Y., Nishina Y., Yorifuji H., Kikuchi T. (2002). Immunolocalization of caveolin-1 and caveolin-3 in monkey skeletal, cardiac and uterine smooth muscles. Cell Struct. Funct..

[24] Kunert-Keil C., Gredes T., Lucke S., Morgenstern S., Mielczarek A., Sporniak-Tutak K., Gedrange T., Spassov A. (2011). Caveolin-1, caveolin-3 and VEGF expression in the masticatory muscles of mdx mice. Folia Histochem. Cytobiol..

[25] Baguma-Nibasheka M., Fracassi A., Costain W.J., Moreno S., Kablar B. (2016). Role of skeletal muscle in motor neuron development. Histol. Histopathol..

[26] Bolger A.M., Lohse M., Usadel B. (2014). Trimmomatic: A flexible trimmer for illumina sequence data. Bioinformatics.

[27] Kim D., Langmead B., Salzberg S.L. (2015). Hisat: A fast spliced aligner with low memory requirements. Nat. Methods.

[28] Pertea M., Kim D., Pertea G.M., Leek J.T., Salzberg S.L. (2016). Transcript-level expression analysis of RNA-seq experiments with HISAT, StringTie and Ballgown. Nat. Protoc..

[29] Kong L., Zhang Y., Ye Z.Q., Liu X.Q., Zhao S.Q., Wei L., Gao G. (2007). CPC: Assess the protein-coding potential of transcripts using sequence features and support vector machine. Nucleic Acids Res..

[30] Sun L., Luo H., Bu D., Zhao G., Yu K., Zhang C., Liu Y., Chen R., Zhao Y. (2013). Utilizing sequence intrinsic composition to classify protein-coding and long non-coding transcripts. Nucleic Acids Res..

[31] Sonnhammer E.L., Eddy S.R., Birney E., Bateman A., Durbin R. (1998). Pfam: Multiple sequence alignments and HMM-profiles of protein domains. Nucleic Acids Res..

[32] Li A., Zhang J., Zhou Z. (2014). PLEK: A tool for predicting long non-coding RNAs and messenger RNAs based on an improved k-mer scheme. BMC Bioinform..

[33] Roberts A., Trapnell C., Donaghey J., Rinn J.L., Pachter L. (2011). Improving RNA-seq expression estimates by correcting for fragment bias. Genome Biol..

[34] Langmead B., Salzberg S.L. (2012). Fast gapped-read alignment with bowtie 2. Nat. Methods.

[35] Roberts A., Pachter L. (2013). Streaming fragment assignment for real-time analysis of sequencing experiments. Nat. Methods.

